# Editorial: Autophagy in inflammation related diseases, volume II

**DOI:** 10.3389/fphar.2023.1273511

**Published:** 2023-08-21

**Authors:** Bo-Zong Shao, Tian Xia, Elena Talero, Yu Bai

**Affiliations:** ^1^ Department of Pharmacy, General Hospital of the Chinese People’s Liberation Army, Beijing, China; ^2^ Department of Gastroenterology, Changhai Hospital, Navy Medical University/Second Military Medical University, Shanghai, China; ^3^ Department of Pharmacology, Faculty of Pharmacy, Universidad de Sevilla, Seville, Spain

**Keywords:** autophagy, inflammation, disease, inflammasome, pharmacology

Autophagy is a vital catabolic mechanism to degrade and recycle long-lived proteins and useless organelles relying on lysosomes (Xu and Wan, 2023). Since its initially discovered and reported in the 1960s, the biological characteristics and functions have been largely investigated. So far, several kinds of autophagy have been uncovered, including classic autophagy (including microautophagy, chaperonemediated autophagy, and macroautophagy) and selective autophagy (including pexophagy, mitophagy, xenophagy, and reticulophagy, etc.) (Shao et al., 2022; Shao et al., 2023; Wang et al., 2023). The general process of autophagy is elucidated, including the formation of phagophores and subsequently autophagosomes with the involvement of autophagy-related genes (ATGs), and the mature of autolysosomes with the integration of autophagosomes and lysosomes (Deretic, 2021; Shao et al., 2022). So far, autophagy has been widely revealed to be closely involved in the pathogenesis and progression of various kinds of inflammation-related diseases. For instance, it was previously reported by studies from us and others that autophagy contributed to alleviate the severity of multiple sclerosis through the suppression of the NLRP3 inflammasome assembly and activation (Shao et al., 2014; Cheng et al., 2020). In addition, autophagy was also shown to be involved in the regulation of other inflammation-related diseases including inflammatory bowel diseases, atherosclerosis, stroke, myocardial infarction, etc., (Sorice, 2022; Yamamoto et al., 2023). Based on such knowledge, we ran another Research Topic about autophagy and inflammation-related diseases entitled “*Autophagy in Inflammation Related Diseases, Volume II*” to collect related studies for the discussion of such issue.

In our Research Topic, six brilliant studies have been collected and officially published in Frontiers in pharmacology. Among them, Wang et al. revealed that aspirin-triggered Resolvin D1 (AT-RvD1) produced an alleviative effect on neuropathic pain through the induction of autophagy-mediated suppression of the NLRP3 inflammasome. In addition, Pei et al. demonstrated that alantolactone attenuated interleukin (IL)-1β-induced inflammatory responses, relieved cartilage degeneration and promoted impaired autophagy via restraining of signal transducer and activator of transcription 3 (STAT3) and nuclear factor (NF)-κB signaling pathways in osteoarthritis. In a review paper, Feng et al. reported that autophagy was involved in the regulation of heparinase-mediated promotion of coagulation disorder and pulmonary fibrosis in acute respiratory distress syndrome (ARDS). In other two review papers, the role of autophagy in the regulation of fibrosis and immunopathogenesis of inflammatory bowel disease was discussed in detail (Macias-Ceja et al. and Li and Law et al.). In addition, Huang et al. demonstrated the hepato-protective role of autophagy in non-alcoholic fatty liver disease (NAFLD).

All in all, our current Research Topic, together with the former one entitled “*Autophagy in Inflammation Related Diseases*,” has collected several latest original studies for the exploration of potential targets taking advantage of autophagy in the treatment of several kinds of inflammation-related diseases. Furthermore, several brilliant review papers have discussed the role of autophagy in several inflammation-related disorders through reviewing and summarizing the previous studies. We believe that our Research Topic would bring new insights in the investigation of autophagy in inflammation-related diseases.
